# Complete genome sequence data of tropical thermophilic bacterium *Parageobacillus caldoxylosilyticus* ER4B

**DOI:** 10.1016/j.dib.2021.107764

**Published:** 2021-12-25

**Authors:** Xin Jie Ching, Nazalan Najimudin, Yoke Kqueen Cheah, Clemente Michael Vui Ling Wong

**Affiliations:** a Biotechnology Research Institute, Universiti Malaysia Sabah, Jalan UMS, 88400 Kota Kinabalu, Sabah, Malaysia; b School of Biological Science, Universiti Sains Malaysia, Persiaran Bukit Jambul, Bayan Lepas, Penang 11900, Malaysia; c Department of Biomedical Science, Faculty of Medicine and Health Sciences, Universiti Putra Malaysia, 43400 UPM Serdang, Selangor Darul Ehsan, Malaysia

**Keywords:** *Parageobacillus caldoxylosilyticus*, Complete whole genome sequence, Thermophile, Thermal stress

## Abstract

*Parageobacillus caldoxylosilyticus*, or previously identified as *Geobacillus caldoxylosilyticus*, is a thermophilic Gram-positive bacterium which can easily withstand growth temperatures ranging from 40 °C to 70 °C. Here, we present the first complete genome sequence of *Parageobacillus caldoxylosilyticus* ER4B which was isolated from an empty oil palm fruit bunch compost in Malaysia. Whole genome sequencing was performed using the PacBio RSII platform. The genome size of strain ER4B was around 3.9Mbp, with GC content of 44.31%. The genome consists of two contigs, in which the larger contig (3,909,276bp) represents the chromosome, while the smaller one (54,250bp) represents the plasmid. A total of 4,164 genes were successfully predicted, including 3,972 protein coding sequences, 26 rRNAs, 91 tRNAs, 74 miscRNA, and 1 tmRNA. The genome sequence data of strain ER4B reported here may contribute to the current molecular information of the species. It may also facilitate the discovery of molecular traits related to thermal stress, thus, expanding our understanding in the acclimation or adaptation towards extreme temperature in bacteria.

## Specifications Table

**Table:** 

Subject	Biology
Specific subject area	Microbiology and Genomics
Type of data	Table Image Figure
How data were acquired	Whole genome sequence of *Parageobacillus caldoxylosilyticus* ER4B was obtained using PacBio RSII
Data format	Raw and Analyzed
Parameters for data collection	Pure culture of strain ER4B was grown in Lennox Broth (LB) at its’ optimal growth temperature 64 °C and the genomic DNA was extracted when the culture reaches mid log phase.
Description of data collection	The genomic DNA was sequenced using PacBio RSII, while subsequent genome assembly and annotation was done using Canu (V1.6) and Prokka (v1.12) respectively.
Data source location	*Parageobacillus caldoxylosilyticus* strain ER4B was previously isolated from an oil palm empty fruit bunch compost in Malaysia on 11th November, 2005, and it was provided by Prof. Dr. Clemente Michael Wong Vui Ling group from Biotechnology Research Institute, Universiti Malaysia Sabah.
Data accessibility	The complete genome sequence of *Parageobacillus caldoxylosilyticus* ER4B has been deposited in NCBI GenBank under accession number CP040553-CP040554.

## Value of the Data

• The data from this work represents the first complete and gapless genome of *Parageobacillus caldoxylosilyticus* as four other genome sequences from the same species deposited in NCBI GenBank are draft genomes.
• Complete whole genome sequence of *Parageobacillus caldoxylosilyticus* ER4B could provide valuable information about thermal adaptation in the bacterium, particularly at high growth temperature.
• Comparative genomics can also be carried out using this genomic data against the genome of different strains, or even different species, and this will definitely contribute in the further development and understanding molecular basis of thermal adaptation in different bacteria.
• The data can be very useful for scientists and students working in the field of microbiology, genomics, and biotechnology in extremophiles, especially thermophiles.

## 1 Data Description

**Fig. 1:**
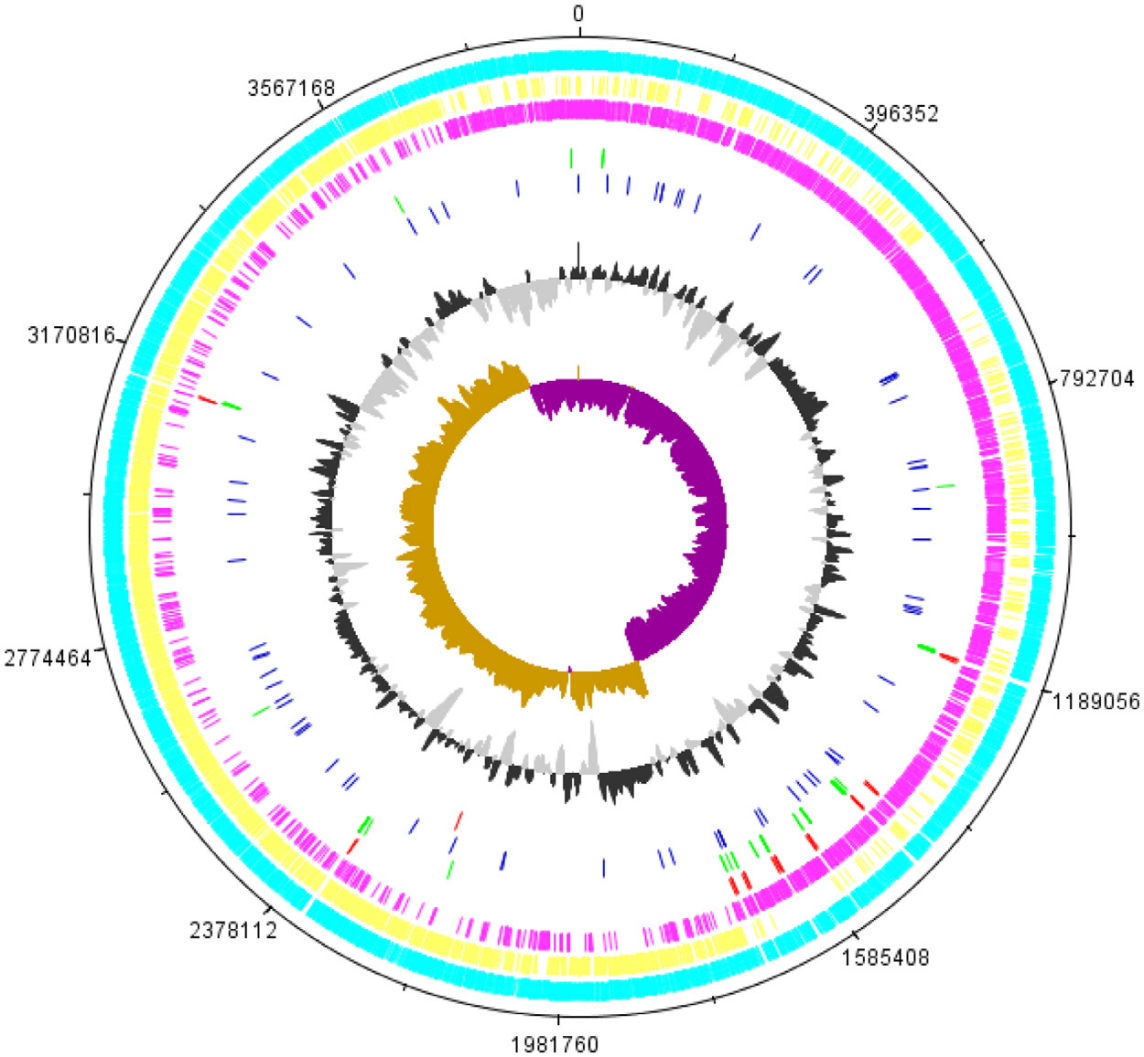
Genome map of ER4B was constructed using DNAPlotter. From the outer track: 1^st^ track represents total annotated genes, 2^nd^ track represents forward CDS, 3^rd^ track represents reverse CDS, 4^th^ track represents rRNA, 5^th^ track represents tRNA, 6^th^ track represents miscellaneous RNA (miscRNA), 7^th^ track represents tmRNA, 8^th^ track represents GC plot, and the last track represents GC skew. Major tick marks interval was set at 1/10^th^ of the overall genome size, which is 396,352bp, so 0 represents both the beginning and the ending of the sequence.

We present the whole genome sequence of *P. caldoxylosilyticus* ER4B that was obtained from PacBio RSII. *P. caldoxylosilyticus* ER4B was previously isolated from an oil palm empty fruit bunch compost, and it would grow optimally at 64 °C in Lennox broth (LB). The genome features of strain ER4B were summarized in Table 1. The assembled genome is approximately 3.96Mbp in size, and comprises of two contigs, where the larger contig represents the chromosome and the smaller contig represents the plasmid. Subsequent genome annotation revealed a total of 4,164 genes were successfully predicted from both chromosome and plasmid, including 3,972 protein coding genes and 192 non-coding RNAs. Among all the predicted CDS, 2,933 of them are genes with known functions, whereas 1,039 are categorized as hypothetical genes. The position of each CDS and RNA genes can be better visualized in the genome map in Fig. 1.

**Table 1:** Genomic features of *P. caldoxylosilyticus* ER4B.

Features	Value
Contigs no.	2
Genome size (bp)	3,963,526
Chromosome size (bp)	3,909,276
Plasmid size (bp)	54,250
GC content (%)	44.32
Total number of genes	4,164 *(C: 4,106 + P: 58)
Protein coding sequences (CDS)	3,972 *(C: 3,914 + P: 58)
Genes with predicted function	2,933 *(C: 2,917 + P: 16)
Hypothetical genes	1,039 *(C: 997 + P: 42)
rRNA	26
tRNA	91
miscRNA	74
tmRNA	1

⁎ C represents chromosome; P represents plasmid.

**Fig. 2:**
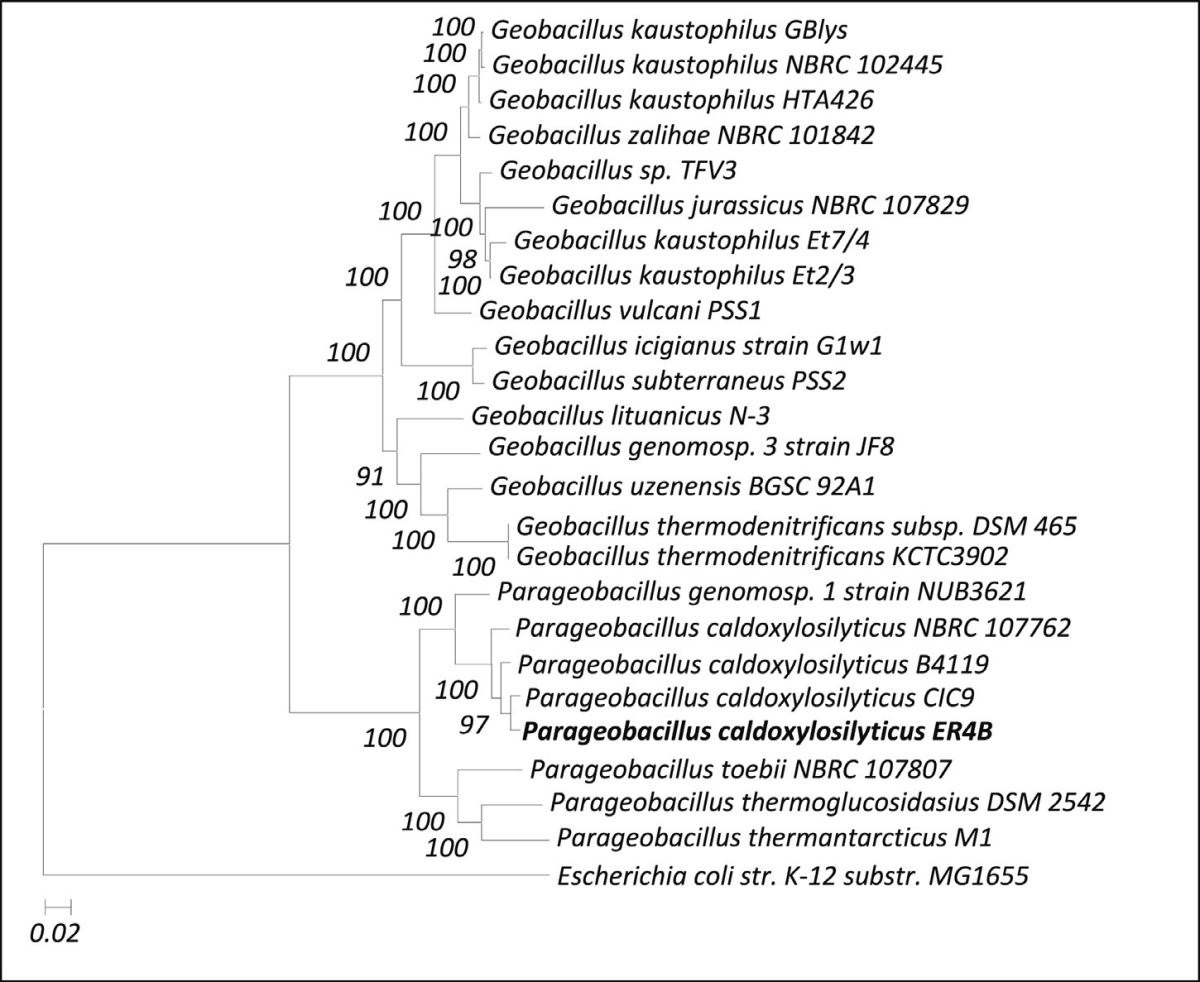
Whole genome phylogenetic tree constructed by PhyloSift, using Maximum Likelihood method based on Generalised Time-Reversible (GTR) model. The tree shows the close relationship between *P. caldoxylosilyticus* ER4B with the close related species, while *E. coli* K-12 substr. MG1655 is included to serve as an outgroup.

The whole genome sequence of strain ER4B was utilized to construct a clearer and more accurate evolutionary relationship with other bacterial whole genomes closely related to *Parageobacillus* and *Geobacillus* through PhyloSift. Fig. 2 clearly depicted that *P. caldoxylosilyticus* CIC9 is the closest strain to ER4B, followed by the other strains from the same species. This reconfirmed the identity of strain ER4B as it is strongly affiliated with other *P. caldoxylosilyticus* strains. It is also noteworthy that *Parageobacillus genomosp.* appeared to be closer to *P. caldoxylosilyticus* as compared to the other three species *Parageobacillus toebii, Parageobacillus thermoglucosidans* and *Parageobacillus thermantarcticus*. Besides, Fig. 2 also showed that *Parageobacillus* and *Geobacillus* were separated at the main node, forming two distinct clades between the two genera as proposed in previous study [1]. This clustering suggests that PhyloSift is able to provide higher phylogenetic resolution and better taxonomy assignment in phylogenetic analysis as compared to the more congurent single-gene phylogenetic analysis.

**Fig. 3:**
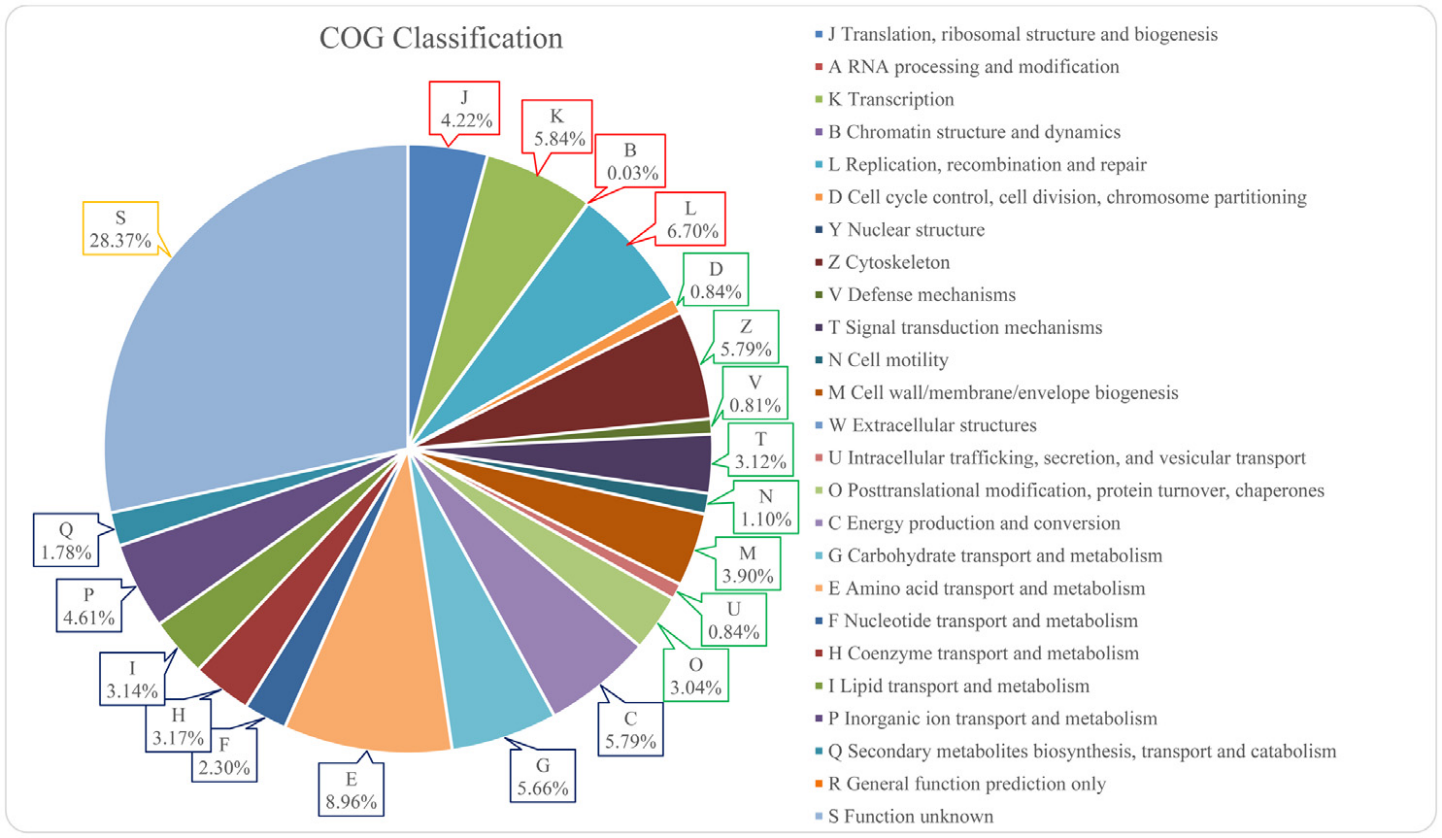
Functional distribution of genes within the *P. caldoxylosilyticus* ER4B genome classified by clusters of orthologous groups (COG). COG in red box refers to major category “information storage and processing”; green box refers to “cellular processes and signaling”; blue box refers to “metabolism”; and yellow bow refers to “poorly characterized”.

The annotated genome was further classified into orthologous group based on their function. 3,819 of the annotated genes were successfully classified into any one of the COG categories. As depicted in Fig. 3, 35.41% of the annotated genes were classified into “Metabolism” major category, followed by “Poorly characterized” with 28.37%, “Cellular processes and signaling” with 19.44%, and “Information storage and processing” with 16.79%. While there was a total of 26 functional COG categories, no eggNOG-annotated genes were found to be categorized under the RNA processing and modification (A), general function prediction only (R), extracellular structures (W), and nuclear structure (Y).

From Fig. 3, it was also clear that most of the annotated genes (28.37%) fell in category S, as these genes encode for hypothetical or novel proteins in which their functions were not readily assigned. Among the remaining genes with assigned functions, 8.96% of the genes which have functions related to amino acid transport and metabolism were classified into category E, making E a category with second highest abundance of genes. The third highest category is L with 6.70%, followed by K (5.84%), C (5.79%), Z (5.79%), and G (5.66%).

Similar to other thermophilic bacteria, strain ER4B is constantly exposed to high growth temperatures. Although group O occupied only 3.04%, it is important for the survival of strain ER4B at high temperatures as many of the heat stress related proteins were categorized in this group. The genome of strain ER4B was found to harbour several genes encoding for heat-related proteins, including GrpE, GroEL, GroES, DnaJ, DnaK, and ClpB [2,^3^,4] as shown in Table 2. These heat shock proteins work in conjunction with one another to prevent protein aggregation at high temperature. Besides, various features responding to heat-induced stress, such as general stress proteins, DNA SOS response proteins, and oxidative stress proteins (Table 2), can also be found in the genome of this bacterium. These proteins would trigger stress responses to prevent or mitigate the cellular damage caused by heat stress, thus crucial in contributing to the thermophilicity in strain ER4B. Interestingly, several copies of cold shock protein B (CspB) was also found in the genome of this thermophilic bacterium [5].

**Table 2:** Number of gene copies for thermal stress related proteins in *P. Caldoxylosilyticus* ER4B.

Thermal stress related proteins	Number of copies
**Cold shock proteins**	
Cold shock protein CspB	3
**Heat shock proteins**	
Chaperone protein DnaJ	1
Chaperone protein DnaK	1
Chaperone protein ClpB	1
Heat shock protein 60 co-chaperone GroES	1
Heat shock protein 60 family chaperone GroEL	1
Heat shock protein GrpE	1
Heat-inducible transcription repressor HrcA	1
small heat shock protein*	6
**Stress proteins**	
General stress protein	9
Universal stress protein	1
**DNA SOS response proteins**	
Putative SOS response-associated peptidase YedK	1
Recombinase A (RecA)	1
LexA repressor	1
**Oxidative stress proteins**	
Catalases	2
Peroxiredoxin and Peroxidase	7
Superoxide dismutase	3
Thioredoxins	9

⁎ Small heat shock proteins include HSP15, HSP18, HSP20, HSP31, and HSP33.

## 2 Experimental Design, Materials and Methods

The genomic DNA of strain ER4B was prepared from cells in the exponential growth phase. DNA extraction was then carried out using Qiagen DNeasy® Blood & Tissue kit (Qiagen, Valencia, CA, USA) according to manufacturer's protocols, with several optimization (personal communication, Yong Sheau Ting) to maximize both quality and quantity of the genomic DNA extracted.

The complete genome of strain ER4B was sequenced using the PacBio RSII instrument (Pacific Biosciences, Menlo Park, CA, USA). The Single Molecule Real Time (SMRT) sequencing was conducted using 20kb SMRT bell templates and DNA Polymerase Binding kit P6-V2 on top of PacBio RSII system. The raw sequencing data obtained was then proceeded with reads correction, trimming, and *de novo* assembly using Canu v1.6 [6]. Subsequently, the assembled genome was annotated using Prokka v1.12 [7], and the complete genome map was constructed using DNAPlotter [8]. The annotated genome of strain ER4B was then used for the construction of the phylogenetic tree using PhyloSift [9]. Furthermore, the annotated genome was further distributed into clusters of orthologous groups (COGs) based on functional annotation using eggNOG-mapper [10].

## Ethics Statement

This work did not involve any animals or human subjects. The manuscript represents the author's original work which has not been published elsewhere.

## CRediT Author Statement

**Clemente Michael Vui Ling Wong, Nazalan Najimudin, Yoke Kqueen Cheah and Xin Jie Ching:** Conceptualization, Methodology; **Xin Jie Ching:** Data curation, Writing- Original draft preparation; **Clemente Michael Vui Ling Wong:** Supervision; **Xin Jie Ching and Clemente Michael Vui Ling Wong:** Writing - Reviewing and Editing.

## Declaration of Competing Interest

The authors declare that they have no known competing financial interests or personal relationships which have or could be perceived to have influenced the work reported in this article.

## Appendix Supplementary materials

Image, application 1
